# Melatonin promotes adventitious root formation in apple by promoting the function of *MdWOX11*

**DOI:** 10.1186/s12870-020-02747-z

**Published:** 2020-11-26

**Authors:** Jiangping Mao, Chundong Niu, Ke Li, Shiyue Chen, Muhammad Mobeen Tahir, Mingyu Han, Dong Zhang

**Affiliations:** 1 College of Horticulture, Northwest Agriculture & Forestry University, Yangling, 712100 China; 2 College of Life Science, Northwest Agriculture & Forestry University, Yangling, 712100 China

**Keywords:** Activation of adventitious roots, Adventitious roots, Apple rootstocks, *MdWOX11*, Melatonin, Transgenic plantlets

## Abstract

**Background:**

Melatonin (MT) is important for plant growth and development; however, it is not known whether MT is involved in apple adventitious root (AR) development. In this study, we treated *Malus prunifolia* (MP) at four different stages of AR development, and analyzed the level of the endogenous hormones MT, auxin (IAA), zeatin-riboside (ZR), abscisic acid (ABA), and gibberellins (GA_1 + 3_) in all four treatment groups and the untreated control group. The expression of MT, IAA biosynthesis, transport and signal transduction, the cell cycle, and root development related genes were quantified by RT-qPCR. The function of *MdWOX11* was analyzed in transgenic apple plants.

**Results:**

The promotion of AR development by MT was dependent on the stage of AR induction between 0 and 2 d in apple rootstocks. MT-treatment increased the level of IAA and crosstalk existed between MT and IAA during AR formation. The expression of *MdWOX11* was induced by MT treatment and positively regulated AR formation in apple. Furthermore, transgenic lines that overexpressed *MdWOX11* lines produced more ARs than ‘GL3’. Phenotypic analysis indicated that *MdWOX11* overexpression lines were more sensitive to exogenous MT treatment than ‘GL3’, suggesting that *MdWOX11* regulates AR formation in response to MT in apple rootstock*.*

**Conclusions:**

MT promotes AR formation mainly during the AR induction stage by inducing IAA levels and upregulating *MdWOX11*.

**Supplementary Information:**

The online version contains supplementary material available at 10.1186/s12870-020-02747-z.

## Background

Apple (*Malus domestica*) is a major commercial fruit tree that is cultivated globally and apple fruits have a high nutritional and economic value. *Malus prunifolia* (MP) is widely known as the easiest-rooting apple rootstock. It offers advantages such as good graft compatibility, cold resistance, salt and alkali tolerance, and disease and insect resistance. Adventitious roots (ARs) induction from stem basal tissues is a major step in the vegetative propagation of apple rootstocks. ARs are post-embryonic roots that emerge from non-root organs, and AR primordia arise from interfascicular cambium cells adjoining phloem cells [1, 2]. The processes required for AR formation have been studied in different plants, including rice [3], *Arabidopsis* [4, 5], and poplar [6]; however, methods for improving AR formation in apple have not been studied.

Melatonin (MT; N-acetyl-5-methoxytryptamine) is a well-known hormone in animal and was initially discovered in plants by two groups of workers in 1995 [7, 8]. Previous studies have shown that MT functions as a regulatory signal in plants [9], and is important for the growth of roots, shoots, explants, and stress responses [10–15]. The relationship between MT and AR formation has mainly been studied in herbaceous plants; for example, the exogenous application of MT promoted adventitious rooting in tomato and rice [16, 17], but the mechanism how MT regulates AR formation remains to be elucidated in woody plants such as apple. AR formation can be categorised as a four-stage process [18–21], and the stage at which MT is important for AR development remains unknown. In this study, we observed that MT promoted AR formation at early stages of AR induction and initiation. Previous studies have demonstrated the relationship between MT and other plant hormones such as auxin (IAA), cytokinin (CK), gibberellins (GA), abscisic acid (ABA) [22]: Treatment with MT caused the CK levels to increase during non-biological stress in the plant [23], MT also contributed to increasing the content of active GAs such as GA_3_ and GA_4_ [24], and exogenous MT application led to a decrease in the content of ABA [23]. However, the relationship between MT and these hormones during adventitious rooting remains to be determined. Potentially, MT acts as a growth-promoting compound by increasing the level of IAA, IAA synthesis and polar IAA transport [25–27]. Most studies have analyzed the ability of MT to stimulate root and shoot growth, in a similar way to IAA [28]. However, the effect of MT on root growth and differentiation is thought to be independent of IAA [29]. In this study, we established that MT–IAA crosstalk plays an important role in AR induction. Still, the role of plant hormone interaction and associated signaling networks during AR formation is incompletely understood in apple rootstock.

The genes involved in MT biosynthesis, such as *TDC*, *SNAT*, *HIOMT* and *ASMT*, are induced by MT [30–34], *MzSNAT5* regulates MT synthesis in the mitochondria of apple [33], and overexpression of *ASMT* increases MT production in *Arabidopsis thaliana* [34]. In this study, the expression of MT and auxin-related genes, such as *AUXIN RESPONSE FACTORS* (*ARFs*) and *PINFORMED* (*PIN*) genes, were analyzed in apples. In addition, *WUSCHEL-RELATED HOMEOBOX GENE 11* (*WOX11*) functions in crown root emergence and development [35] and AR development in Arabidopsis [36], but in woody plants, the regulation of *WOX11* during AR development is poorly understood. Furthermore, it is unknown whether AR formation in transgenic apple plants that overexpress *MdWOX11* is regulated by exogenous MT.

Currently, the mechanisms via which AR formation is regulated by MT is not well characterized in apple rootstock. In this study, we showed that exogenous MT induced AR formation at the early stages of AR induction and initiation by increasing IAA synthesis, transport, and the expression of signaling-related genes. Apple plantlets treated with MT in tissue culture showed an increase in the expression of root development-related genes, and therefore, an increase in the number of ARs. Furthermore, we demonstrated that overexpression of *MdWOX11* promoted the emergence and development of ARs, and treatment with exogenous MT induced AR development in the transgenic apple that overexpressed *MdWOX11*. The results of this study provide insights into the mechanism of how MT regulates AR formation in apple rootstock.

## Results

**Fig. 1:**
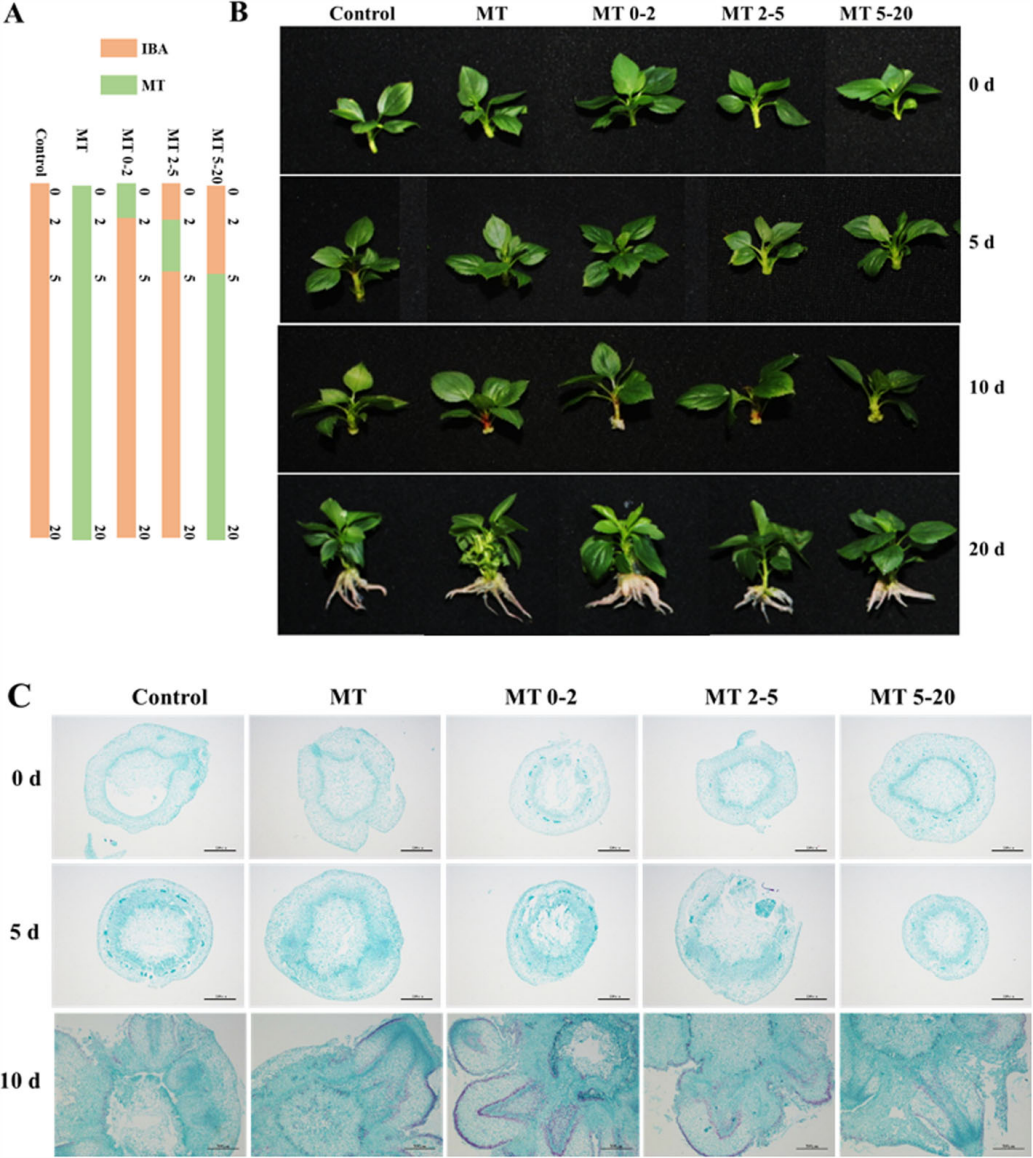
Morphological and anatomical observations of AR development in paraffin-embedded sections from the experimental samples generated in the study at three sampling times: 0 d, 3 d, and 10 d. Five treatment groups were analyzed: **a** The control group in which apple tissue culture plantlets were continuously cultured on root-inducing medium containing 3.45 μM IBA; the MT group was continuously cultivated on 3.45 μM IBA and 1.29 μM MT, and based on different times of MT-treatment, the MT treatment group was also divided into three groups of MT0–2, MT2–5 and MT5–20; **b** Observations of morphological AR formation in the five treatment groups at 0 d, 5 d, 10 d and 20 d; **c** Anatomical observations of AR formation in the five treatment groups at 0 d, 5 d and 10 d

The aim of this study was to identify the precise time at which MT promotes AR formation in tissue-culture plantlets of MP apple rootstocks. In this study, 0–2 d represents the stage of AR induction, 2–5 d represents AR initiation, and 5–20 d covers the stages of AR primordium formation and AR emergence in MP. The study consisted of five different treatment groups: MT, MT0–2, MT2–5, MT5–20, and one control group (Fig. 1a). No morphological changes were evident in any groups up to 5 d; however, ARs emerged from basal stem parts at 10 d (Fig. 1b). At 20 d, the greatest number of ARs was observed in the MT0–2 group, MT2–5 and MT5–20 groups produced more ARs than the control and MT group at 20 d, no significant difference was observed in the number of ARs among these groups (Fig. 1b). To observe the anatomy of stems at different stages of AR formation, sections were made from paraffin-embedded samples and were viewed using light microscopy. On 0 day, cross-sections of the samples revealed the existence of competent cells. Still, mitotic cambial cell division was observed at 5 d, cell divisions were visible in the compactly arranged cells. AR appeared in sections from stem bases cultured in the medium for 10 days (Fig. 1c). No AR formation was observed in MP treated for 20 d with the auxin inhibitors N-1-naphthylphthalamic acid (NPA) or triiodobenoic acid (TIBA), but ARs were observed following MT treatment. All phenotypes were summarised in Figure S1.

**Fig. 2:**
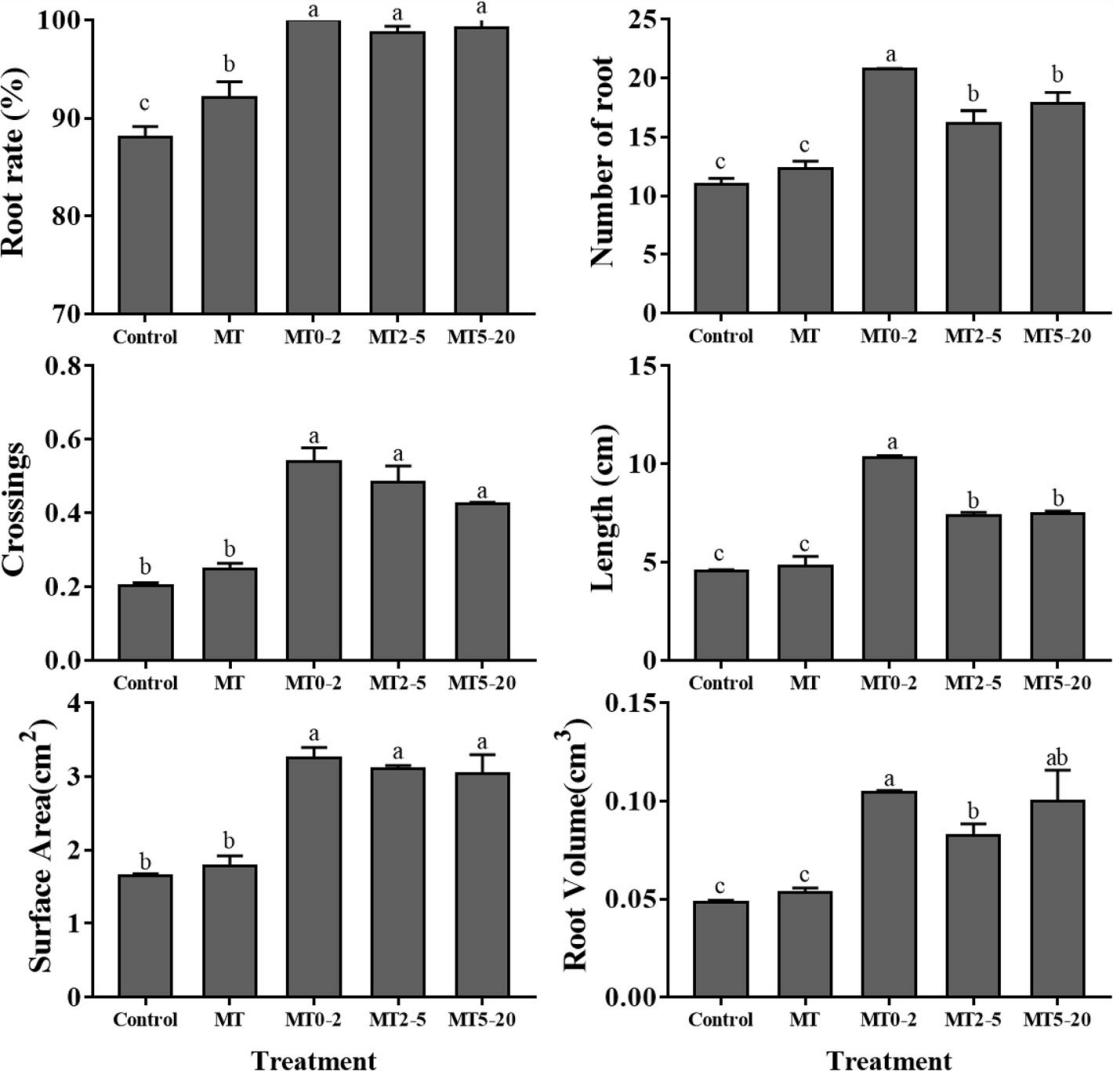
Morphological parameters of AR formation in tissue culture plantlets of *Malus prunifolia*. Tissue culture plantlets were divided into five groups: Control, MT, MT0–2, MT2–5, MT5–20. The number of ARs, and their length, surface area and volume were measured in the five treatment groups. Values represent the mean ± SE for three biological replicates; letters indicate significant differences between means (*P* < 0.05)

We also measured the rooting rate, number of AR, crossings, root length, root volume, and root surface area in all five treatment groups, and the data were consistent with the phenotypes of AR formation. All measured parameters were higher in the MT0–2 group than in other groups, and the minimum number of ARs and values for other root parameters were observed in the control group (Fig. 2). The results showed that MT mainly promoted AR formation at 0–2 d, during the AR induction stage.

**Fig. 3:**
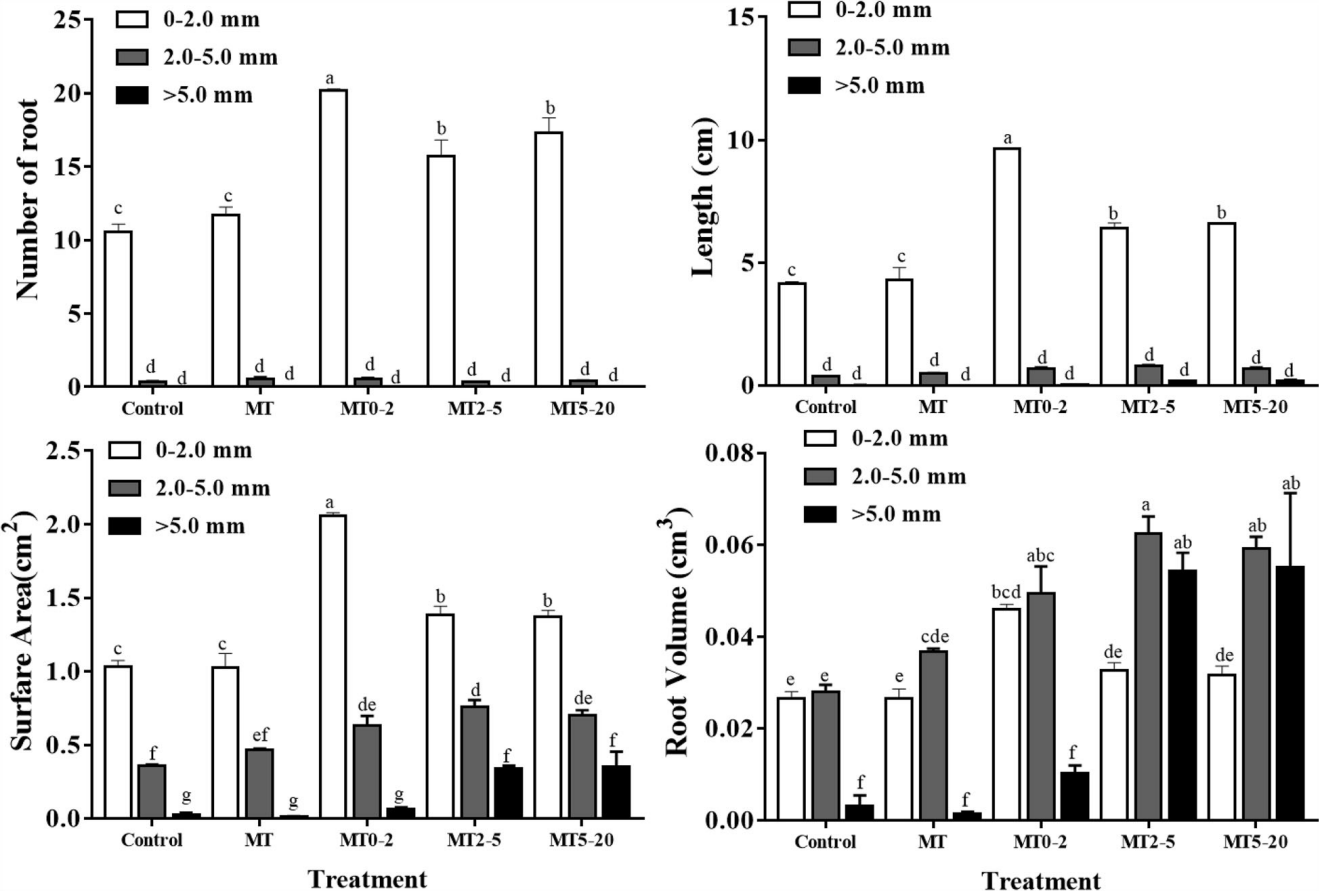
Morphological parameters of AR formation in tissue culture plantlets of *Malus prunifolia* for different categories of root diameter, according to the number, length, surface area, and volume of root. The ARs were classified into three groups based on root diameter: 0–2.0 mm, 2.0–5.0 mm and > 5.0 mm

Based on their diameter, ARs were classified into three groups: 0–2.0 mm, 2.0–5.0 mm and > 5.0 mm. According to AR number, length and surface area, the 0–2.0 mm category contained the greatest percentage of the total; however, for root volume, the 2.0–5.0 mm category was the largest for all groups (Fig. 3). The MT0–2 group contained the greatest number of ARs in the 0–2.0 mm class, which was twice as many as in the control group (Fig. 3). We conclude that most ARs were fine roots (0–2.0 mm).

**Fig. 4:**
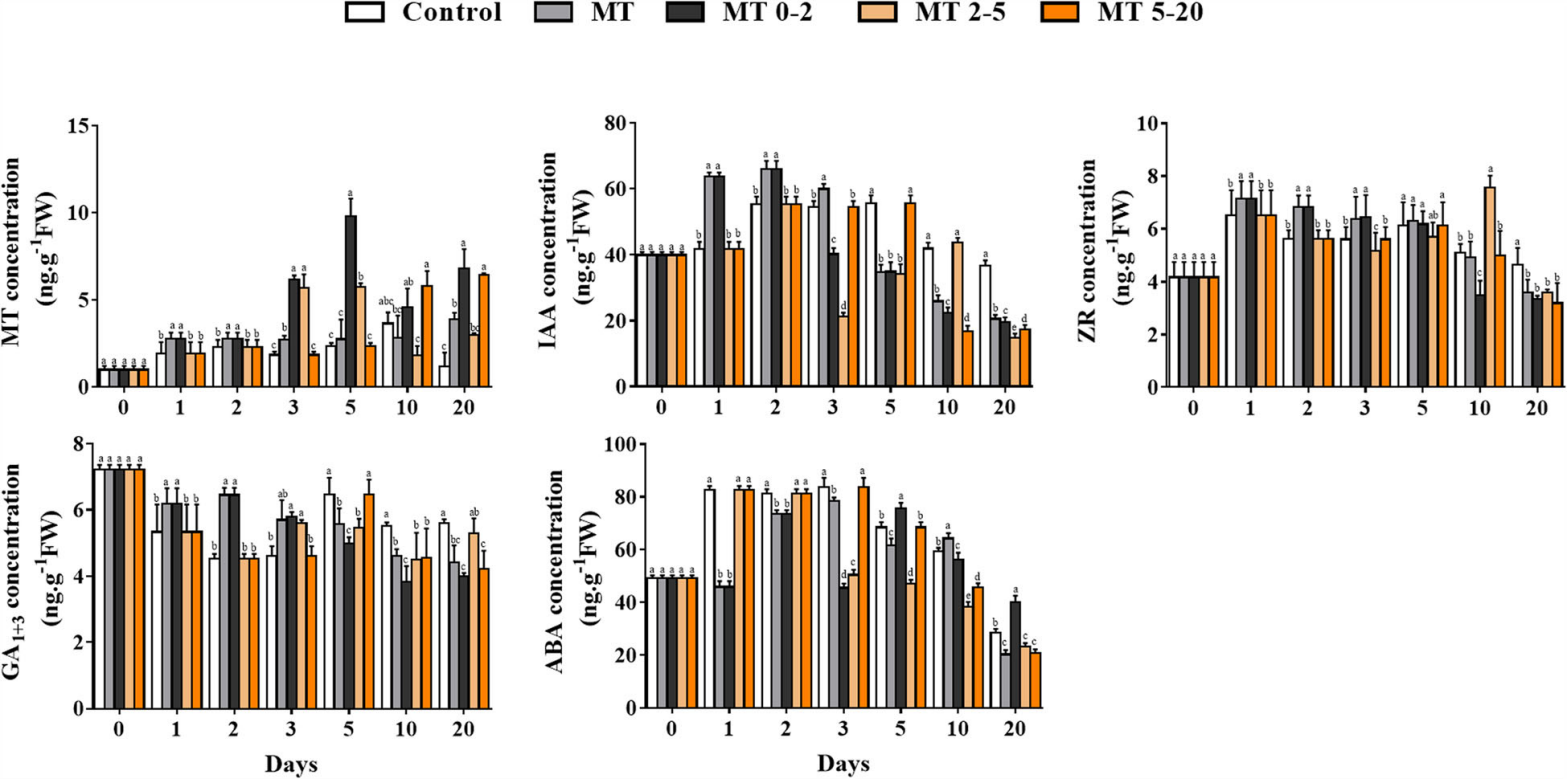
The effect of exogenous application of MT for different lengths of time on the contents of MT, ZR, IAA, GA_1 + 3_, and ABA at different stages of AR development in five treatment groups of *Malus prunifolia*

The levels of the hormones MT, IAA, ZR, GA_1 + 3_ and ABA were analyzed in MP tissue culture plantlets after treatment with MT. The MT content was higher in the MT0–2 group than in other treatment groups during the early AR developmental stage and reached a peak at 5 d in the MT0–2 group. In the MT and MT0–2 treatments, the levels of IAA, ZR and GA_1 + 3_ were higher than those in the control group during AR induction at 1 d and 2 d, but the levels were lower in the MT0–2 group than other groups at 10 d. The level of ABA responded opposite to that of IAA, ZR and GA_1 + 3_ in the treatments (Fig. 4).

**Fig. 5:**
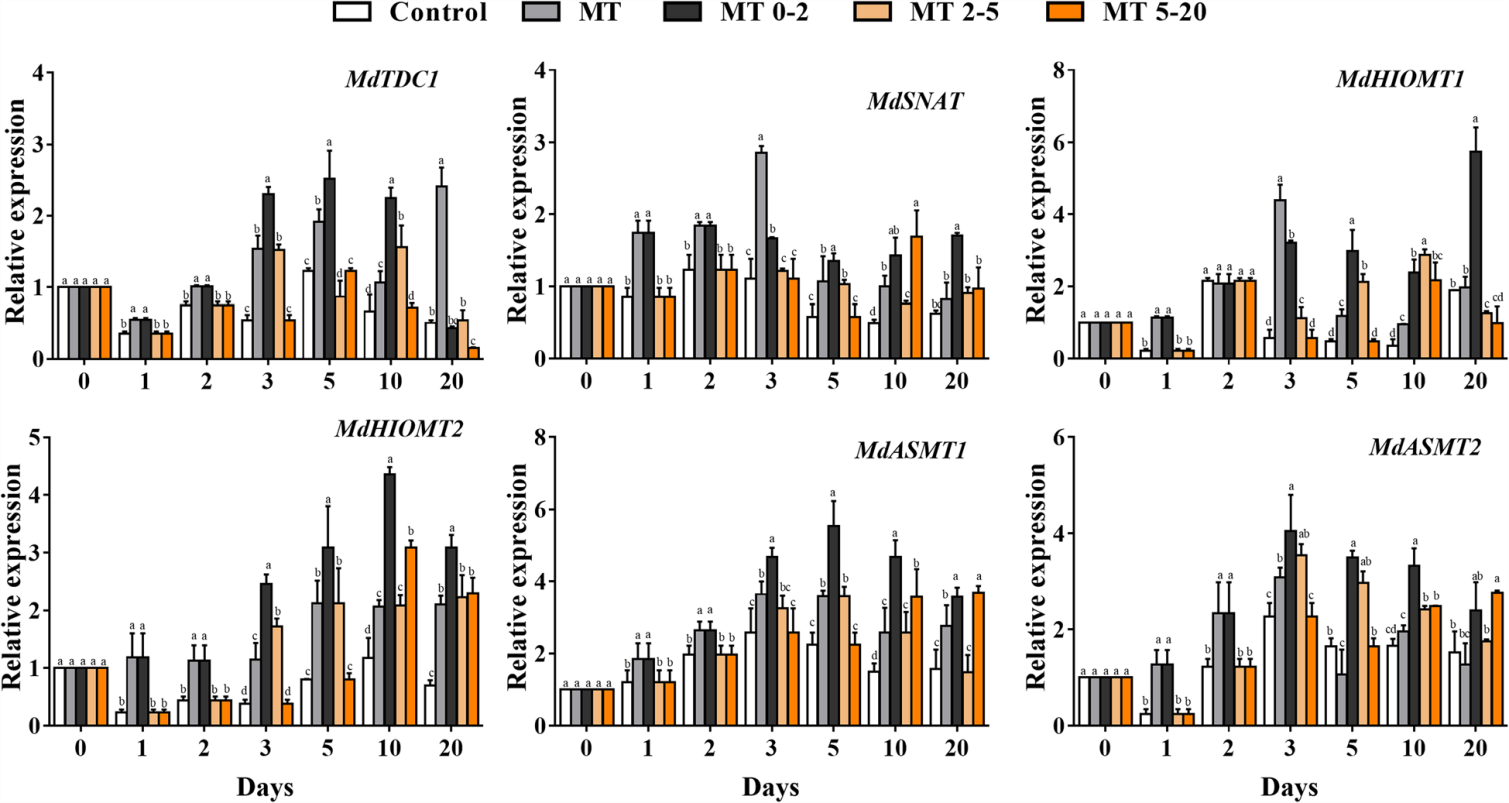
The effect of exogenous MT treatment on the relative expression of genes involved in MT synthesis at different stages of AR development in five treatment groups of *Malus prunifolia*

Furthermore, the expression levels of MT synthesis related genes were analyzed during AR formation. Except for 0 and 20 d, the expression levels of *MdTDC1*, *MdHIOMT2*, *MdASMT1* and *MdASMT2* genes, which are involved in MT synthesis, were higher in the MT0–2 groups than those of other groups, and the expression levels of *MdSNAT* and *MdHIOMT1* were higher in MT0–2 than those of other groups at 1 d, 5 d and 20 d. These results suggested that MT treatment induced the expression of MT synthesis related genes (Fig. 5).

**Fig. 6:**
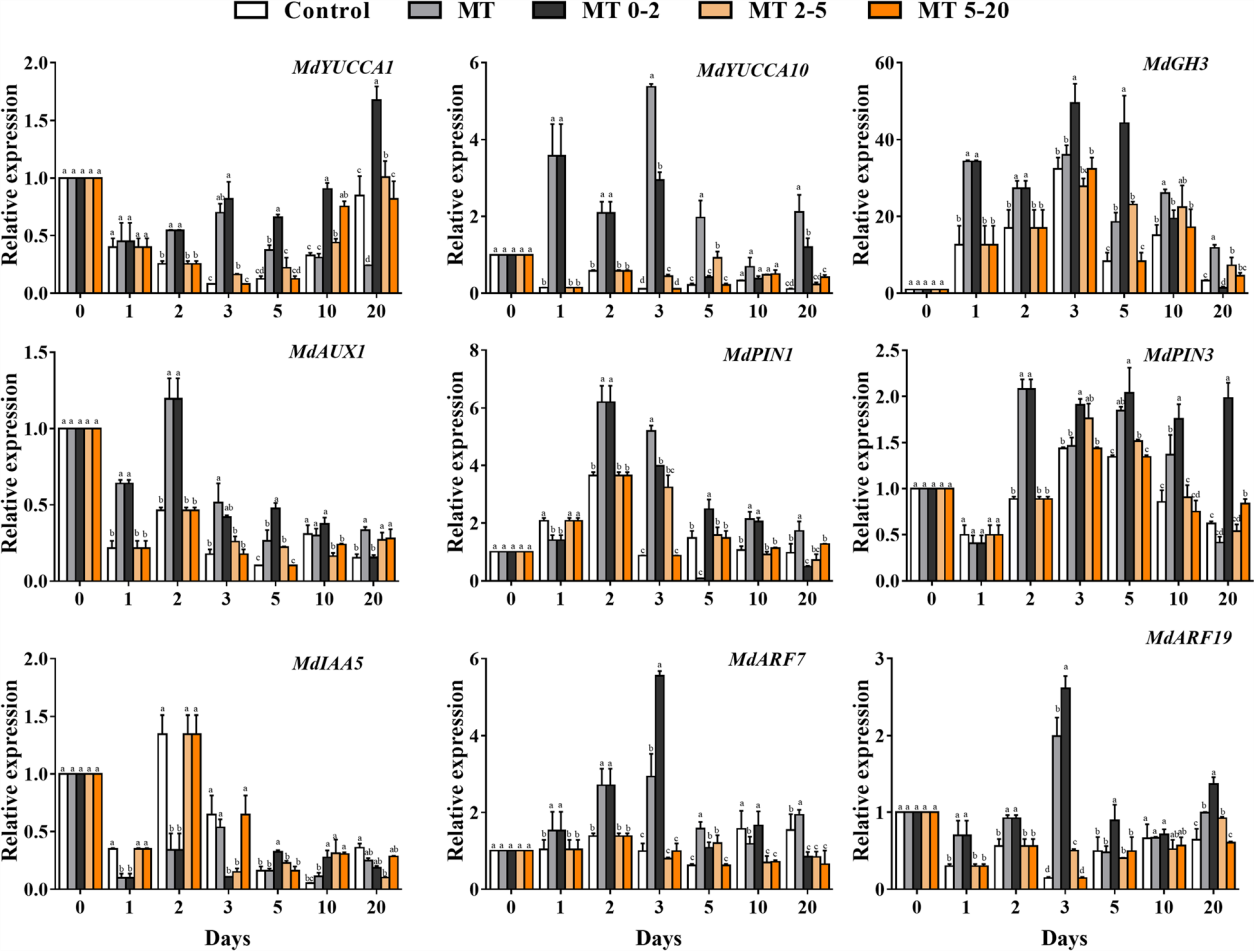
The effect of exogenous MT treatment on the relative expression of genes involved in auxin biosynthesis, transport, and signal transduction at different stages of AR development in five treatment groups of *Malus prunifolia*

To determine whether an interactive effect between MT and IAA existed, we measured the expression of genes related to IAA biosynthesis and signal transduction. The expression of *MdYUCCA1*, *MdYUCCA10*, *MdARF7*, and *MdARF19* were higher in the MT0–2 treatment group than that in other groups at 1 d, 2 d and 3 d. The expression level of the IAA transport-related genes *MdAUX1*, *MdPIN1*, and *MdPIN3* were also higher in the MT0–2 than in other groups at 2 d; however, expression of the IAA signal transduction gene *MdIAA5* was lower following MT0–2 treatment than in other treatments during the AR induction stage (Fig. 6).

**Fig. 7:**
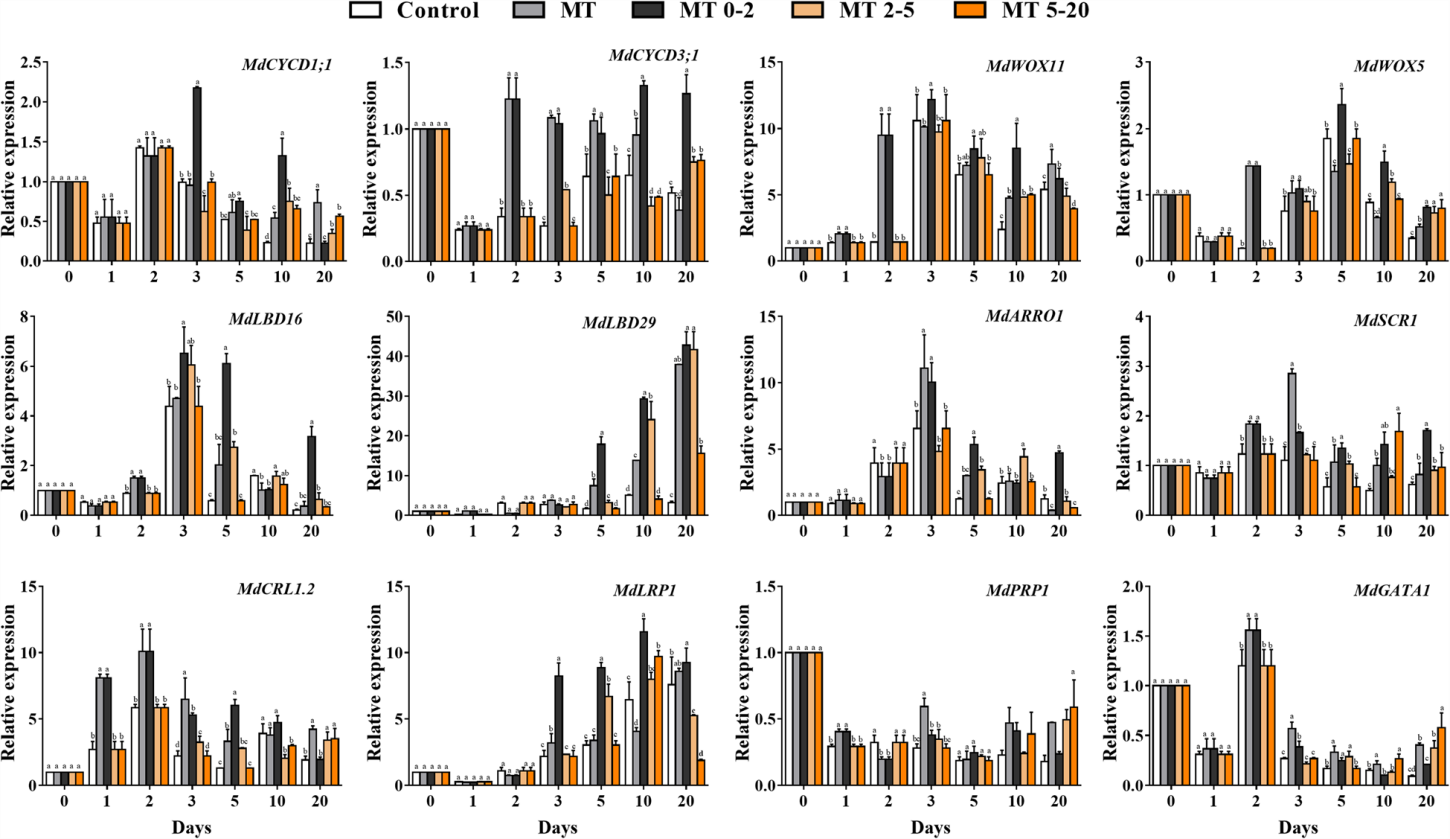
The effect of exogenous MT treatment on the relative expression of genes involved in the cell recycle and root development at different stages of AR formation in five treatment groups of *Malus prunifolia*

To investigate whether MT affects cell division, the expression of the cell-cycle related genes *MdCYCD1;1* and *MdCYCD3;1* were analyzed, these genes were more highly expressed in MT0–2 at 3 d and 10 d. Therefore, we conclude that the application of MT promoted AR formation in apple, and RT-qPCR analysis showed that the expression of root development-related genes was higher at most sampling time points in response to MT treatment. We observed that among all root development-related genes, *MdWOX11* expression following MT treatment was 5.6 times higher than that in control plants at 2 d (Fig. 7). This suggests that *MdWOX11* probably plays an important role in AR induction in response to MT treatment.

**Fig. 8:**
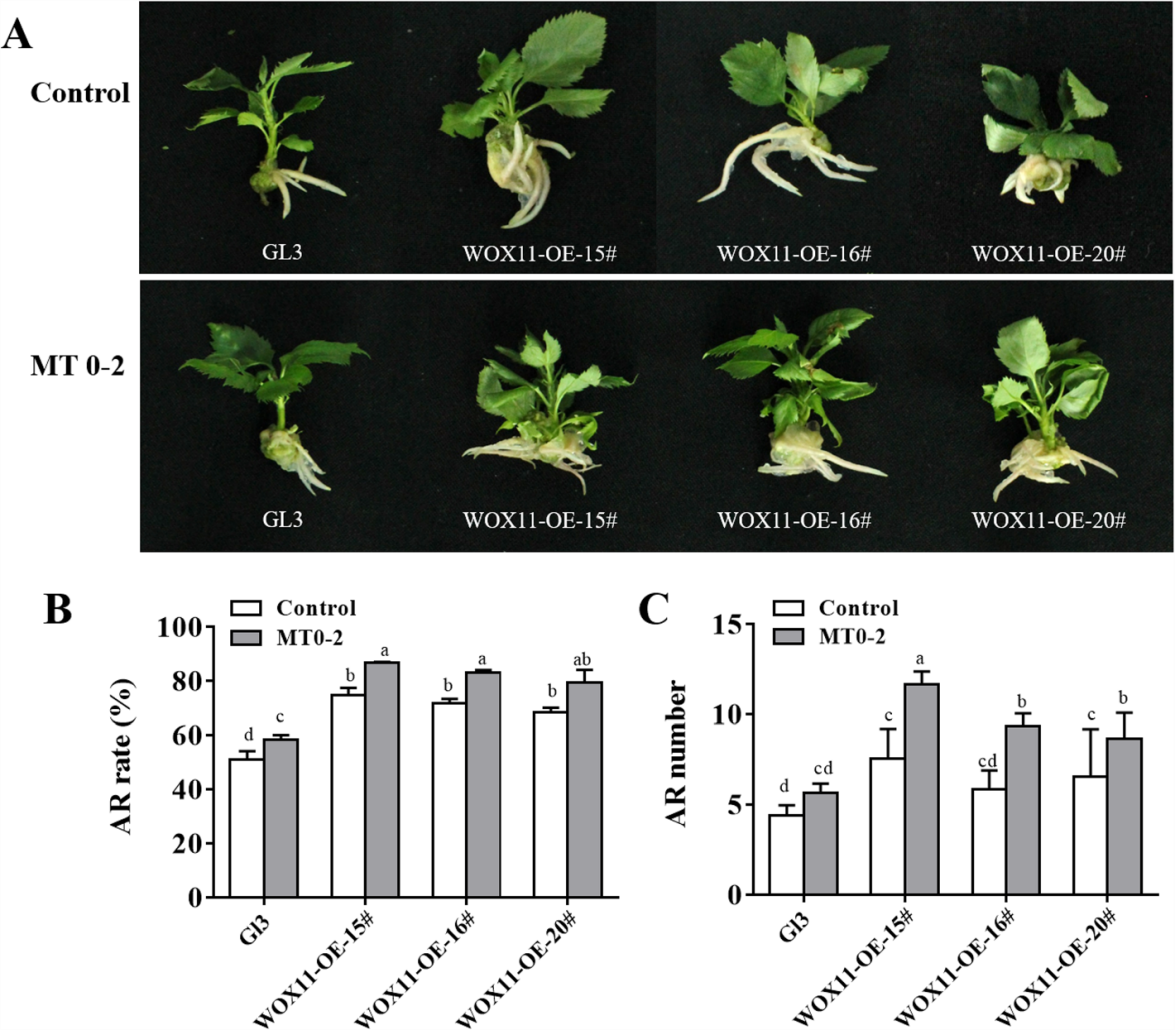
**a** Morphological observations, **b** AR rate, and **c** AR number during AR formation in transgenic tissue-culture plantlets overexpressing of *MdWOX11* (*35S::WOX11-OE*) and wild type ‘GL3’ after treatment with 3.45 μM IBA as the control. Another group was treated with MT for 0–2 d (MT0–2 group)*,* the results represent measurements after culturing the controls and MT0–2 group for 20 d

The expression of *MdWOX11* was induced by IBA treatment (Fig. 7). We generated the overexpression (OE) transgenic lines *MdWOX11-OE15#, 16#* and *20#* in ‘GL3’, and confirmed the level of overexpression *MdWOX11* transgenic lines (Figure S2). To confirm whether *MdWOX11* transgenic lines exhibited an enhanced response to MT signaling, wild-type and transgenic apple plantlets growing in tissue culture were either treated with 3.45 μM IBA as a control or with MT for 0–2 d (MT0–2). More ARs were observed in the MT0–2 group than in the control group, both the overexpressing *MdWOX11* transgenic lines and ‘GL3’, and the *MdWOX11* overexpressing lines produced more ARs than ‘GL3’ (Fig. 8a). Overexpression of *MdWOX11* also caused an increase in the rate of ARs (Fig. 8b). Furthermore, *MdWOX11* overexpressing plants were more sensitive to exogenous MT treatment than wild type (Fig. 8a-c), which indicates that *MdWOX11* induced AR formation in response to MT treatment.

## Discussion

AR formation is the key to vegetative propagation and previous studies have divided AR formation from tissue culture plantlets into four stages: induction, initiation, primordium formation, and emergence [18–21]. In this study, 0–2 d represents the stage of AR induction, 2–5 d represents AR initiation, and 5–20 d covers the stages of AR primordium formation and AR emergence in MP. MT affects many biological processes, including plant growth, flowering and stress responses, and although MT promotes AR development in tomato [16], no studies have been conducted in woody plants. Therefore, to determine when and how MT can promote AR formation, we treated MP plants with MT at different times. The phenotypes of the five treatment groups showed that there was no difference between plants in the MT2–5 and MT5–20 groups, but the MT0–2 group produced more ARs than other groups (Figs. 1, 2 and 3), which demonstrates that MT promotes AR formation mainly during the AR induction stage at 0–2 d.

The effect of interaction between MT and IAA during root development is unclear. Some studies have suggested that a low concentration of exogenous MT can induce an increase in the endogenous IAA levels in plants, and it is believed that the promoting effect of MT on growth might be caused by this increase in IAA content [25]. However, other research has shown that the regulation of root growth and differentiation by MT was independent of IAA [29]. To analyse whether MT treatment can elevate the IAA content and enhance IAA signaling, we measured the IAA content in plants in the five MT treatments. The IAA content mainly increased during AR induction after MT application, but decreased during the AR initiation and AR emergence stages (Fig. 4). This might reflect that IAA plays an important role during the early stage of root development [37–39]. During AR formation, MP plantlets were treated with IAA inhibitors and MT (Figure S1). NPA and TIBA treatment inhibited AR formation, NPA and TIBA have the function of inhibiting IAA polar transport. We conclude that exogenous MT treatment promoted AR formation to influence the IAA distribution in the AR zone. Furthermore, it suggests that IAA may be downstream of MT to induce AR formation. However, RT-qPCR data suggest that MT is involved in IAA signaling pathways, and MT treatment can induce the expression of IAA biosynthesis, transport and signal transduction-related genes (Fig. 6). Therefore, MT potentially promotes AR induction by increasing IAA levels and IAA signaling. Previous research has shown that MT-treated plants have increased CK levels during non-biological stress [23], and MT also increased in the content of active GAs such as GA_3_ and GA_4_ [24]. The observed increase in the levels of GA and ZR following MT treatment also suggests that there is a link between MT and ZR or GA_1 + 3_.

*WOX11* regulates AR formation in response to IAA in Arabidopsis [36], and some studies have demonstrated that *WOX11* is induced by IAA and positively induces the expression of *LATERAL ORGAN BOUNDARIES DOMAIN16* (*LBD16*) and *LBD29* at an early stage of AR development [40]. However, little is known concerning the function of *MdWOX11* in woody plants such as apple, including the morphological changes that occur in transgenic apple plants in response to MT during AR formation. In this study, we generated transgenic apple plants that expressed *35S::WOX11-OE*. Their phenotype demonstrated that *MdWOX11* is a positive regulator of AR activation and that MT treatment of these *MdWOX11* overexpressing plants increased AR development (Fig. 8). Therefore, the induction of *MdWOX11* and its related genes might represent a possible mechanism by which MT promotes AR formation. This is the first study to use *MdWOX11* transgenic lines to investigate the role of *MdWOX11* in response to MT during AR induction. Collectively, the data indicate that *MdWOX11* promotes AR formation in response to MT, and provide insights into the molecular mechanisms that underlie the induction of ARs by MT.

## Conclusions

Melatonin promotes adventitious root formation mainly at the stage of AR induction by increasing IAA levels and activating the function of *MdWOX11*. The results represent the potential to improve AR formation to accelerate the asexual reproduction of apple rootstock that is difficult to root.

## Methods

### Explant growth conditions and MT treatments

Tissue culture plantlets of MP apple rootstock were grown in tissue culture in the Yangling (108°04′E, 34°16′N), China, and were used as plantlets for AR formation. The plantlets of MP were imported from Aomori in Japan and were propagated by asexual reproduction. The tissue culture plantlets of MP were split into five groups, and plants in all groups were treated simultaneously for 20 d. Control plants were cultured on a root-inducing medium containing half-strength MS supplemented with 3.45 μM indole-3-butyric acid (IBA) to promote root formation. The second group plants were cultivated on the medium with half-strength MS supplemented with 1.29 μM MT and 3.45 μM IBA and was designated as the MT treatment group. The third group was transferred to root-inducing medium after cultivation on MT medium for 2 d and was called MT 0–2. The fourth group (MT 2–5) was transferred to MT medium after culture on root-inducing medium for 2 d, then it was transferred to root-inducing medium after culture on MT medium for 3 d. The fifth group was transferred to MT medium after cultivation on root-inducing medium for 5 d, and was called MT5–20. The composition of the different medium used for this study was listed in Supplemental Table S1. Samples were harvested from all five groups at 0 d, 1 d, 2 d, 3 d, 5 d, 10 d and 20 d (even though some samples were collected before MT-treatment). In total, 3150 cuttings, consisting of 630 cuttings from each of the five groups were harvested, which in turn, consisted of 90 cuttings sampled at each sampling point. Samples were collected from the basal part of the stems, including the AR formation zone (approximately 0.5 cm). The plants in the NPA treatment group were continuously cultivated in 10 μM NPA and 1.29 μM MT for 20 d, and the TIBA-treated plants were continuously cultivated in 10 μM TIBA and 1.29 μM MT for 20 d, the control was the same as above. Overexpression of *MdWOX11* transgenic apple (*35S::MdWOX11-OE*) and ‘GL3’ were separated into two groups: one group was continuously cultured on root-inducing medium, which was served as the controls, and the other group was transferred to root-inducing medium after culturing in MT medium for 2 d.

### Anatomical observations and morphological measurements

Anatomical observations were carried out according to previously described protocols [1, 41, 42]. The morphological parameters measured included: AR rate, AR length and mean AR number for each cutting [43]. Also, an Epson Expression 10000XL scanner (LA l600 scanner, Canada) was used to analyse other root related indicators. In total, 90 cuttings were analyzed, with 90 cuttings from each group collected at each sampling point. The harvested samples were immediately frozen in liquid nitrogen and stored at − 80 °C for hormone and expression analysis.

### Measurement of hormone levels

Samples for hormone extraction were harvested at different time points from all five treatment groups. Hormones were purified and extracted according to previously described procedures [44]. Three biological replicates for each group at each sampling point were analyzed. The enzyme-linked immunosorbent assay (ELISA) technique was used to detect and analyse the level of hormones [44].

### Extraction of RNA and synthesis of cDNA

CTAB-based extraction method was used to isolate total RNA [45], and the total RNA integrity was tested by electrophoresis of the samples on 2% agarose gels. Prime Script RT Reagent Kit with gDNA Eraser (TaKaRa Bio, Shiga, Japan) was used to synthesize cDNA.

### RT-qPCR analyses

MT, IAA synthesis, transport and signal transduction, the cell cycle and root development related genes expression were quantified by RT-qPCR. The gene full names and abbreviations, the MDP annotations in apple, as well as homologous proteins and species on which the identification of the proteins in apple was based, were listed in Supplemental Table S2. Primer design was based on previous research [43], and all gene-specific primers for the analyzed genes were listed in Supplemental Table S3.

RT-qPCR was performed according to published methods [46]. The apple *EF-α* gene was used to normalize expression. Each sample contained three biological replicates and three technical replicates. The analyzed genes relative expression were calculated by the 2^−ΔΔCt^ method [47].

### Statistical analysis

SPSS11.5 software (SPSS, Chicago, IL, USA) was used to analysis significant differences, significance differences among each sampling time point and treatment were determined using (ANOVA). SigmaPlot12.0 (Systat Software, Inc.) was used to generate figures.

## Supplementary Information

**Additional file 1:** **Figure S1.** Morphological observations of AR formation in tissue culture plantlets of *Malus prunifolia* at 20 d. IBA group were continuously cultivated in 3.45 μM IBA and 1.29 μM MT, NPA group were continuously cultivated in 10 μM NPA and 1.29 μM MT, TIBA group were continuously cultivated in 10 μM TIBA and 1.29 μM MT. **Figure S2.** Identification of DNA level of in overexpression *MdWOX11* transgenic lines, marker was 2000 bp, wild type is named as WT, there are three lines in overexpression *MdWOX11* transgenic lines, they are *MdWOX11OE-15#*, *MdWOX11OE-16#*, *MdWOX11OE-20#*, H_2_O was set as negative control. **Table S1**. Composition of medium. **Table S2**. The gene name (the abbreviation and full name) and the apple MDP number, as well as the species and protein of the homologue on which the apple protein was based on. **Table S3**. Sequence of primers used for expression analysis, F for the former primer, R for the rear primer, MDP number of gene and length of primers.

## Data Availability

All data generated or analyzed during this study are included in this published article and its supplementary information files.

## Abbreviations

MT: Melatonin
AR: Adventitious root
MP: *Malus prunifolia*
IAA: Auxin
CK: Cytokinin
GA: Gibberellins
ABA: Abscisic acid
ELISA: Enzyme-linked immunosorbent assay
WOX11: WUSCHEL-RELATED HOMEOBOX GENE 11
IBA: Indole-3-butyric acid
LBD16: LATERAL ORGAN BOUNDARIES DOMAIN16

## References

[1] Naija S, Elloumi N, Jbir N, Ammar S, Kevers C (2008). Anatomical and biochemical changes during adventitious rooting of apple rootstocks MM 106 cultured *in vitro*. C R Biol.

[2] Jásik J, Klerk GJD (1997). Anatomical and ultrastructural examination of adventitious root formation in stem slices of apple. Biol Plant.

[3] Jiang W, Zhou S, Zhang Q, Song H, Zhou DX, Zhao Y (2017). Transcriptional regulatory network of WOX11 is involved in the control of crown root development, cytokinin signals, and redox in rice. J Exp Bot.

[4] Gutierrez L, Mongelard G, Floková K, Păcurar DI, Novák O, Staswick P, Kowalczyk M, Păcurar M, Demailly H, Geiss G (2012). Auxin controls Arabidopsis adventitious root initiation by regulating jasmonic acid homeostasis. Plant Cell.

[5] Hu X, Xu L (2016). Transcription factors WOX11/12 directly activate WOX5/7 to promote root primordia initiation and organogenesis. Plant Physiol.

[6] Rigal A, Yordanov YS, Perrone I, Karlberg A, Tisserant E, Bellini C, Busov VB, Martin F, Kohler A, Bhalerao R (2012). The AINTEGUMENTA LIKE1 homeotic transcription factor PtAIL1 controls the formation of adventitious root primordia in poplar. Plant Physiol.

[7] Dubbels R, Reiter RJ, Klenke E, Goebel A, Schnakenberg E, Ehlers C, Schiwara HW, Schloot W (1995). Melatonin in edible plants identified by radioimmunoassay and by high performance liquid chromatography-mass spectrometry. J Pineal Res.

[8] Hattori A, Migitaka H, Iigo M, Itoh M, Yamamoto K, Ohtanikaneko R, Hara M, Suzuki T, Reiter RJ (1995). Identification of melatonin in plants and its effects on plasma melatonin levels and binding to melatonin receptors in vertebrates. Biochem Mol Biol Int.

[9] Park WJ (2011). Melatonin as an endogenous plant regulatory signal: debates and perspectives. J Plant Biol.

[10] Sarropoulou V, Dimassi-Theriou K, Therios I, Koukourikou-Petridou M (2012). Melatonin enhances root regeneration, photosynthetic pigments, biomass, total carbohydrates and proline content in the cherry rootstock PHL-C (Prunus avium x Prunus cerasus). Plant Physiol Biochem.

[11] Li C, Liang B, Chang C, Wei Z, Zhou S, Ma F (2016). Exogenous melatonin improved potassium content in Malus under different stress conditions. J Pineal Res.

[12] Arnao MB, Hernández-Ruiz J (2007). Melatonin promotes adventitious- and lateral root regeneration in etiolated hypocotyls of *Lupinus albus L*. J Pineal Res.

[13] Byeon Y, Back K (2014). An increase in melatonin in transgenic rice causes pleiotropic phenotypes, including enhanced seedling growth, delayed flowering, and low grain yield. J Pineal Res.

[14] Małgorzata M, Posmyk, Krystyna M, Janas (2009). Melatonin in plants. Acta Physiol Plant.

[15] Park S (2012). Back K. melatonin promotes seminal root elongation and root growth in transgenic rice after germination. J Pineal Res.

[16] Wen D, Gong B, Sun S, Liu S, Wang X, Wei M, Yang F, Li Y, Shi Q (2016). Promoting roles of melatonin in adventitious root development of *Solanum lycopersicum L* by regulating auxin and nitric oxide signaling. Front Plant Sci.

[17] Liang C, Li A, Yu H, Li W, Liang C, Guo S, Zhang R, Chu C (2017). Melatonin regulates root architecture by modulating Auxin response in Rice. Front Plant Sci.

[18] Atkinson JA, Rasmussen A, Traini R, Voss U, Sturrock C, Mooney SJ, Wells DM, Bennett MJ (2014). Branching out in roots: uncovering form, function, and regulation. Plant Physiol.

[19] Klerk GJD, Arnholdt-Schmitt B, Lieberei R, Neumann KH (1997). Regeneration of roots, shoots and embryos: physiological, biochemical and molecular aspects. Biol Plant.

[20] Legue V, Rigal A, Bhalerao RP (2014). Adventitious root formation in tree species: involvement of transcription factors. Physiol Plant.

[21] Klerk G-J (2002). Rooting of microcuttings. Theory and practice. In Vitro Cell Dev Biol Anim.

[22] Arnao MB, Hernandez-Ruiz J (2018). Melatonin and its relationship to plant hormones. Ann Bot.

[23] Jing Z, Yi S, Zhang X, Du H, Xu B, Huang B (2017). Melatonin suppression of heat-induced leaf senescence involves changes in abscisic acid and cytokinin biosynthesis and signaling pathways in perennial ryegrass ( *Lolium perenne* L.). Environ Exp Bot.

[24] Hai-Jun Z, Na Z, Rong-Chao Y, Li W, Qian-Qian S, Dian-Bo L, Yun-Yun C, Sarah W, Bing Z, Shuxin R (2015). Melatonin promotes seed germination under high salinity by regulating antioxidant systems, ABA and GA? Interaction in cucumber (Cucumis sativus L.). J Pineal Res.

[25] Chen Q, Qi WB, Russel J, Wei W, Wang BM (2009). Exogenously applied melatonin stimulates root growth and raises endogenous indoleacetic acid in roots of etiolated seedlings of *Brassica juncea*. J Plant Physiol.

[26] Wang Q, An B, Wei Y, Reiter RJ, Shi H, Luo H, He C (2016). Melatonin regulates root meristem by repressing auxin synthesis and polar auxin transport in *Arabidopsis*. Front Plant Sci.

[27] Arnao MB, Hernández-Ruiz J (2017). Growth activity, rooting capacity, and tropism: three auxinic precepts fulfilled by melatonin. Acta Physiol Plant.

[28] Marino B (2006). Arnao, Hernández-Ruiz J. the physiological function of melatonin in plants. Plant Signal Behav.

[29] Ramo´ n Pelagio-Flores, Edith Mun˜ oz-Parra, Randy Ortiz-Castro, pez-Bucio. JL (2012). Melatonin regulates Arabidopsis root system architecture likely acting independently of auxin signaling. J Pineal Res.

[30] Ma Q, Zhang T, Zhang P, Wang Z-Y (2016). Melatonin attenuates postharvest physiological deterioration of cassava storage roots. J Pineal Res.

[31] Sangkyu P, Da-Eun L, Hyunki J, Yeong B, Young-Soon K, Kyoungwhan B (2013). Melatonin-rich transgenic rice plants exhibit resistance to herbicide-induced oxidative stress. J Pineal Res.

[32] Yeong B, Hyoung Yool L, Ok Jin H, Hye-Jung L, Kyungjin L, Kyoungwhan B (2015). Coordinated regulation of melatonin synthesis and degradation genes in rice leaves in response to cadmium treatment. J Pineal Res.

[33] Wang L, Feng C, Zheng X, Guo Y, Zhou F, Shan D, Liu X, Kong J (2017). Plant mitochondria synthesize melatonin and enhance the tolerance of plants to drought stress. J Pineal Res.

[34] Zuo B, Zheng X, He P, Lin W, Lei Q, Feng C, Zhou J, Li Q, Han Z, Kong J (2014). Overexpression of *MzASMT* improves melatonin production and enhances drought tolerance in transgenic *Arabidopsis thaliana* plants. J Pineal Res.

[35] Zhao Y, Hu Y, Dai M, Huang L, Zhou DX (2009). The WUSCHEL-related homeobox gene WOX11 is required to activate shoot-borne crown root development in rice. Plant Cell.

[36] Liu J, Sheng L, Xu Y, Li J, Yang Z, Huang H, Xu L (2014). WOX11 and 12 are involved in the first-step cell fate transition during de novo root organogenesis in *Arabidopsis*. Plant Cell.

[37] Laskowski M (2013). Lateral root initiation is a probabilistic event whose frequency is set by fluctuating levels of auxin response. J Exp Bot.

[38] Mao J-P, Zhang D, Zhang X, Li K, Liu Z, Meng Y, Lei C, Han M-Y (2018). Effect of exogenous indole-3-butanoic acid (IBA) application on the morphology, hormone status, and gene expression of developing lateral roots in *Malus hupehensis*. Sci Hortic.

[39] Himanen K (2002). Auxin-mediated cell cycle activation during early lateral root initiation. Plant Cell.

[40] Liu B, Wang L, Zhang J, Li J, Zheng H, Chen J, Lu M (2014). WUSCHEL-related Homeobox genes in Populus tomentosa: diversified expression patterns and a functional similarity in adventitious root formation. BMC Genomics.

[41] Qiyun XU, Chai F, Xincheng AN, Han S (2012). Production method for paraffin section of invasive species of *Bemisia Tabaci*. Plant Dis Pests.

[42] Yang JP (2006). Improvement of traditional paraffin section preparation methods. J Biol.

[43] Mao J, Zhang D, Meng Y, Li K, Wang H, Han M (2018). Inhibition of adventitious root development in apple rootstocks by cytokinin is based on its suppression of adventitious root primordia formation. Physiol Plant.

[44] Dobrev PI, Kamínek M (2002). Fast and efficient separation of cytokinins from auxin and abscisic acid and their purification using mixed-mode solid-phase extraction. J Chromatography A.

[45] Gambino G, Perrone I, Gribaudo I (2008). A rapid and effective method for RNA extraction from different tissues of grapevine and other woody plants. Phytochem Anal.

[46] Li G, Ma J, Tan M, Mao J, An N, Sha G, Zhang D, Zhao C, Han M (2016). Transcriptome analysis reveals the effects of sugar metabolism and auxin and cytokinin signaling pathways on root growth and development of grafted apple. BMC Genomics.

[47] Livak KJ, Schmittgen TD (2001). Analysis of relative gene expression data using real-time quantitative PCR and the 2(^−ΔΔCt^) method. Methods..

