# Supplementary material for: Melatonin promotes adventitious root formation in apple by promoting the function of MdWOX11
Source: BMC Plant Biol. 2020 Nov 26;20:536. doi: 10.1186/s12870-020-02747-z (PMC7690037; doi:10.1186/s12870-020-02747-z)
Supplement: Supplementary file 1 — Additional file 1: Figure S1. Morphological observations of AR formation in tissue culture plantlets of Malus prunifolia at 20 d. IBA group were continuously cultivated in 3.45 μM IBA and 1.29 μM MT, NPA group were continuously cultivated in 10 μM NPA and 1.29 μM MT, TIBA group were continuously cultivated in 10 μM TIBA and 1.29 μM MT. Figure S2. Identification of DNA level of in overexpression MdWOX11 transgenic lines, marker was 2000 bp, wild type is named as WT, there are three lines in overexpression MdWOX11 transgenic lines, they are MdWOX11OE-15#, MdWOX11OE-16#, MdWOX11OE-20#, H2O was set as negative control. Table S1. Composition of medium. Table S2. The gene name (the abbreviation and full name) and the apple MDP number, as well as the species and protein of the homologue on which the apple protein was based on. Table S3. Sequence of primers used for expression analysis, F for the former primer, R for the rear primer, MDP number of gene and length of primers. [file 12870_2020_2747_MOESM1_ESM.docx]

Figure S1 Morphological observations of AR formation in tissue culture plantlets of *Malus prunifolia* at 20 d. IBA group were continuously cultivated in 3.45 μM IBA and 1.29 μM MT, NPA group were continuously cultivated in 10 μM NPA and 1.29 μM MT, TIBA group were continuously cultivated in 10 μM TIBA and 1.29 μM MT.


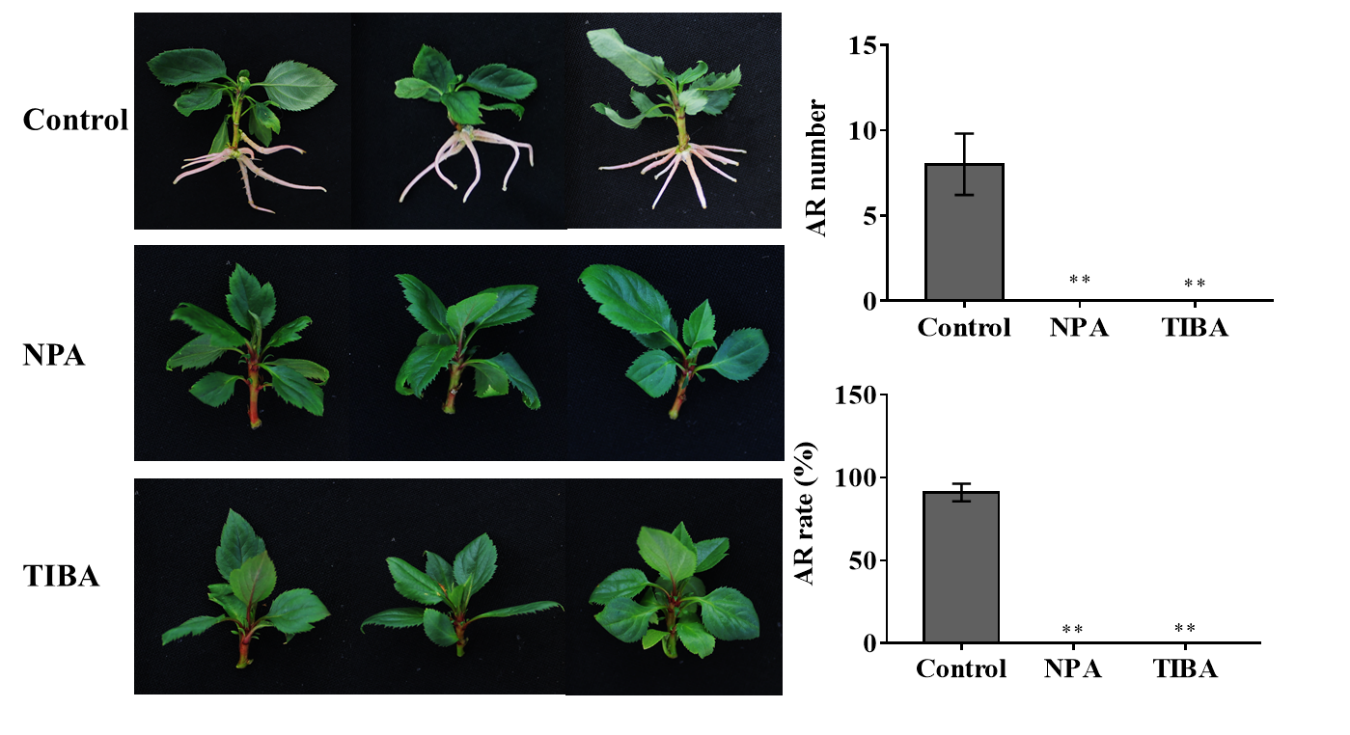


Figure S2 Identification of DNA level of in overexpression *MdWOX11* transgenic lines, marker was 2000bp, wild type is named as WT, there are three lines in overexpression *MdWOX11* transgenic lines, they are *MdWOX11OE-15#*, *MdWOX11OE-16#*, *MdWOX11OE-20#*, H_2_O was set as negative control.


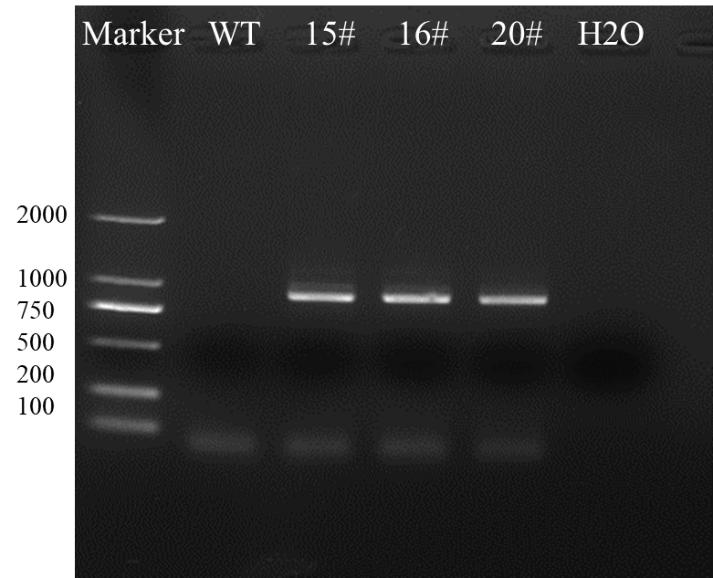


**Table S1**. Composition of medium.

| **Treatment** | **Medium** | **IBA**  **（mg/L）** | **Melatonin**  **（mg/L）** | **Sugar**  **（g/L）** | **Agar**  **（g/L）** |
| --- | --- | --- | --- | --- | --- |
| **Control** | **1/2 MS** | **0.7** | **0** | **20** | **8** |
| **MT** | **1/2 MS** | **0.7** | **0.3** | **20** | **8** |

**Table S2**. The gene name (the abbreviation and full name) and the apple MDP number, as well as the species and protein of the homologue on which the apple protein was based on.

| **Abbreviation name** | **Full name** | **Apple MDP number** | **Source：species** | **protein：Locus** |
| --- | --- | --- | --- | --- |
| TDC1 | tryptophan decarboxylase 1 | MDP0000170244 | Manihot esculenta | ANN44945 |
| SNAT | serotonin N-acetyltransferase | MDP0000187484 | Manihot esculenta | ANN44947 |
| HIOMT1 | methyltransferase/hydroxyindole-O-methyltransferase 1 | MDP0000656929 | Manihot esculenta | ANN44952 |
| HIOMT2 | methyltransferase/hydroxyindole-O-methyltransferase 1 | MDP0000208322 | Manihot esculenta | ANN44953 |
| ASMT1 | N-acetylserotonin-Omethyltransferase 1 | MDP0000851398 | Manihot esculenta | ANN44949 |
| ASMT2 | N-acetylserotonin-Omethyltransferase 2 | MDP0000283649 | Manihot esculenta | ANN44950 |
| YUCCA1 | Probable indole-3-pyruvate monooxygenase YUCCA1 | MDP0000295839 | Arabidopsis thaliana | YUC1_ARATH |
| YUCCA10 | flavin containing monooxygenase YUCCA10-like | MDP0000582079 | Arabidopsis thaliana | ARR1_ARATH |
| GH3 | Grecthenhagen-3 | MDP0000811081 | Arabidopsis thaliana | XP_028953638 |
| AUX1 | auxin influx carrier 1 | MDP0000749280 | Arabidopsis thaliana | ABF47279 |
| PIN1 | Pin-Formed 1 | MDP0000138035 | Arabidopsis thaliana | OAP14866 |
| PIN3 | Pin-Formed 3 | MDP0000497581 | Arabidopsis thaliana | OAP15729 |
| IAA5 | indole-3-acetic acid inducible 5 | MDP0000213864 | Arabidopsis thaliana | OAP14637 |
| ARF7 | Auxin Response Factors 7 | MDP0000185253 | Arabidopsis thaliana | OAP17736 |
| ARF19 | Auxin Response Factors 19 | MDP0000876321 | Arabidopsis thaliana | OAP07000 |
| WOX5 | WUSCHEL-related homeobox 5 | MDP0000213910 | Arabidopsis thaliana | OAO92914 |
| WOX11 | WUSCHEL-related homeobox 11 | MDP0000136426 | Arabidopsis thaliana | YUC1_ARATH |
| SHR | SHORT-ROOT | MDP0000840369 | Arabidopsis thaliana | YUC10_ARATH |
| LBD16 | Lateral Organ Boundaries Domain 16 | MDP0000145761 | Arabidopsis thaliana | CAA67308 |
| LBD29 | Lateral Organ Boundaries Domain 29 | MDP0000131964 | Arabidopsis thaliana | ABR09074 |
| CYCD1;1 | cell cycle regulator | MDP0000231873 | Arabidopsis thaliana | NP_001331451 |
| CYCD3;1 | cell cycle regulator | MDP0000135392 | Arabidopsis thaliana | OAP15151 |
| ARRO1 | Adventitious Rooting Related Oxygenase 1 | MDP0000820500 | Apple | XP_015641726 |
| CRL1 | Charged amino acid-rich leucine zipper 1 | MDP0000299673 | Arabidopsis thaliana | AAG35177 |
| GATA1 | GATA transcription factor 1 | MDP0000190038 | Arabidopsis thaliana | NP_189047 |
| LRP1 | Lateral root primordium (LRP) protein-related 1 | MDP0000312744 | Arabidopsis thaliana | AAA87790 |
| SCR1 | GRAS family transcription factor | MDP0000203826 | Arabidopsis thaliana | SCRA_ARATH |
| PRP1 | putative pre-mRNA-splicing factor ATP-dependent RNA helicasePRP1 | MDP0000538670 | Poplar | AAT67078 |

**Table S3**. Sequence of primers used for expression analysis, F for the former primer, R for the rear primer, MDP number of gene and length of primers.

| **Gene** | **MDP** | **Sequence 5’-3’** | **length** |
| --- | --- | --- | --- |
| TDC1-F | MDP0000170244 | GTGCTTGCATTTGTCCCGAG | 20 |
| TDC1-R | MDP0000170244 | CCCAAAGGCAACAACAGTCG | 20 |
| SNAT-F | MDP0000187484 | ATCTGCACGGTTGCGTCATA | 20 |
| SNAT-R | MDP0000187484 | ACCGGAAGTGGAAGTGCAAA | 20 |
| HIOMT1-F | MDP0000656929 | TTCTCATAGGAGCTTAGTTGCTC | 23 |
| HIOMT1-R | MDP0000656929 | AATGCTCGTCACTCCAGTCG | 20 |
| HIOMT2-F | MDP0000208322 | GTCGATGACTATTTTGTAGTGCTAA | 25 |
| HIOMT2-R | MDP0000208322 | AATGATCGTCACTCCAGTCGT | 21 |
| ASMT1-F | MDP0000851398 | AGGGACGATGACGATGACAAC | 21 |
| ASMT1-R | MDP0000851398 | AGACTGCAACACAACGACCA | 20 |
| ASMT2-F | MDP0000283649 | ATTGTGCCGGGTTTCCTCAT | 20 |
| ASMT2-R | MDP0000283649 | ACATGGTATCAGAGCAGGTTCA | 22 |
| YUCCA1-F | MDP0000295839 | AAGATGAGCGACCCATTCCC | 20 |
| YUCCA1-R | MDP0000295839 | AGGAAAACGAGGGTCAAGCC | 20 |
| YUCCA10-F | MDP0000582079 | CAAGTATCCGATCATTGAC | 22 |
| YUCCA10-R | MDP0000582079 | CCTCTTATGCTGCCTATT | 22 |
| GH3-F | MDP0000811081 | GTGGGGGCAATCTGAAAATCAA | 22 |
| GH3-R | MDP0000811081 | TGAAAAACCTCACTCTTGCGT | 21 |
| AUX1-F | MDP0000749280 | GCAGCACAGAAGCAATCAGAGG | 22 |
| AUX1-R | MDP0000749280 | CCGCCATGCCAGAGGAAGTT | 22 |
| PIN1-F | MDP0000138035 | ATCAACCGCTTCGTCGCTCTC | 22 |
| PIN1-R | MDP0000138035 | AGGCAGAGTGGAGACGGAGAA | 22 |
| PIN3-F | MDP0000497581 | ACAATTCAACAGCAGCAGCA | 22 |
| PIN3-R | MDP0000497581 | CGGTCCCACCAAAAACATGG | 21 |
| IAA5-F | MDP0000213864 | CAGCTCTACGCATCACCACCAA | 22 |
| IAA5-R | MDP0000213864 | CCGCCGCTTGCCTTCTTAGTT | 21 |
| ARF7-F | MDP0000185253 | ACGCCACTTGCTTACAACAGGA | 20 |
| ARF7-R | MDP0000185253 | ACGCCATTGTGAGTGCTTCCAT | 20 |
| ARF19-F | MDP0000876321 | ACGGCTCTGTTGCTCCTAACCA | 22 |
| ARF19-R | MDP0000876321 | CGGCTGCTGCTGTTGCTGAA | 24 |
| WOX5-F | MDP0000213910 | TCCGAACGCCGAGCACTGAT | 20 |
| WOX5-R | MDP0000213910 | TGAAGGGTCTCAATCACTCTTGCC | 24 |
| WOX11-F | MDP0000136426 | CCGAAGCCAGAGCAAATCCT | 20 |
| WOX11-R | MDP0000136426 | TTCAAGGCTTGCCTGCAACT | 20 |
| LBD16-F | MDP0000145761 | CGCCAGCAATGTGTCCAAGTT | 20 |
| LBD16-R | MDP0000145761 | GTGAGCCACGCATCCATAAACG | 19 |
| LBD29-F | MDP0000131964 | CGCCAGCAATGTGTCCAAGTTG | 18 |
| LBD29-R | MDP0000131964 | TGGTGAGCCAGTTGAGCCTTTG | 22 |
| CYCD1;1-F | MDP0000231873 | CCAATGTGCTACCACTCCAACTCC | 20 |
| CYCD1;1-R | MDP0000231873 | GGTGATGATGACGATGGCGAAGAT | 21 |
| CYCD3;1-F | MDP0000135392 | GCGGTGGATTGGATGCTGAGAG | 21 |
| CYCD3;1-R | MDP0000135392 | TGAAGTTGGAGGCTGGACAAGAAC | 24 |
| ARRO1-F | MDP0000820500 | GCTCAGACTCTCCACAAAACTACA | 24 |
| ARRO1-R | MDP0000820500 | ATTGGCATTAGGCTCACTTCTCT | 20 |
| CRL1-F | MDP0000299673 | ACCGATTACGTGCCAACTGT | 20 |
| CRL1-R | MDP0000299673 | TTCGTAGCTGGCTTTGCTGA | 22 |
| PRP1-F | MDP0000538670 | GCTGCTCGTGCCATCCTTCAA | 22 |
| PRP1-R | MDP0000538670 | GGCGTTCCTCTAGCTGTCCAAG | 22 |
| GATA1-F | MDP0000190038 | AGATGAGGATGATGCGGAAGATGT | 24 |
| GATA1-R | MDP0000190038 | CTCCTAATTGCGATGATGGATGCT | 24 |
| LRP1-F | MDP0000312744 | GATGGTGATGGTGATGGCGATGTT | 24 |
| LRP1-R | MDP0000312744 | CTGCGGCGTTGTTGGAAGAAGA | 22 |
| SCR1-F | MDP0000203826 | TCCTTATGCGGTGACTCTGTCCAA | 24 |
| SCR1-R | MDP0000203826 | ACTCTTCTTCACTCGTCGGTTCCT | 24 |
| EF-α-F |  | ATTCAAGTATGCCTGGGTGC | 20 |
| EF-α-R |  | CAGTCAGCCTGTGATGTTCC | 20 |
